# Globalizing opposition to pro-environmental institutions: The growth of counter climate change organizations around the world, 1990 to 2018

**DOI:** 10.1371/journal.pone.0315012

**Published:** 2025-01-22

**Authors:** Jared Furuta, Patricia Bromley

**Affiliations:** 1 Center on Philanthropy and Civil Society, Stanford University, Stanford, California, United States of America; 2 Graduate School of Education and Doerr School of Sustainability, Stanford University, Stanford, California, United States of America; University of Jyväskylä: Jyvaskylan Yliopisto, FINLAND

## Abstract

More than two decades of social scientific research has identified the growing network of corporations, think tanks, nonprofits, and advocacy organizations that aim to obstruct climate change action within the United States. Conventional arguments emphasize the role of economic self-interest (e.g., wealthy and powerful corporations) in shaping the rise of an organized “counter climate change movement” that seeks to discredit evidence about anthropogenic climate change and derail solutions to address the problem. In this paper, we track the growth of counter climate change organizations around the world and emphasize the role of reactionary cultural dynamics in driving their emergence. As climate change discourse is infused in more areas throughout society, climate change issues become more salient in the public sphere, generating adversarial grievances, identities, and mobilization among oppositional groups. Drawing on panel logistic regression models for 162–164 countries from 1990 to 2018, we find that counter climate change organizations are most likely to develop in countries with more extensive state policies and structures oriented toward protecting the natural environment, net of a variety of factors that account for a country’s economic interests or its overall capacity to produce domestic associations.

## Introduction

For as long as science has documented anthropogenic climate change, there have been organized efforts to discredit the evidence and block policies aimed at countering it. Such climate denial activism used to have a simple source–economic self-interest. Wealthy conservative philanthropists and powerful corporations have funded a network of think tanks, nonprofits, advocacy organizations, and trade associations that aimed to obstruct climate action through what Brulle [1] calls the “counter climate change movement.” Over a decade of evidence confirms the link between counter climate activism, anti-government conservatism, and corporate financial interests in the United States (US) [1–4].

The existing explanation for the rise of the counter climate change movement as rooted in anti-government economic self-interest works in part, but this focus fails to account for the full scope of anti-climate activity on two fronts. First, the movement is no longer straightforwardly rooted in conservative and economic interests: It is now part of a wider culture war with populist and anti-science dimensions shaped by the more general erosion of the international liberal order [5, 6]. Second, the movement is not limited to the United States: It has gone global. Anti-climate organizations now arise even in countries with relatively limited fossil fuel interests, such as Burkina Faso, New Zealand, or Sweden. By 2022, over 50 countries in the world were home to at least one organization that engages in counter-climate change action. While the United States continues to house most of the world’s counter climate change organizations, explanations that solely emphasize American money and politics no longer fully capture the scope and scale of the movement.

What factors beyond economic self-interest explain the spread of counter climate change organizations around the world over the past several decades? McKie [7–9] points to a growing internationalization of the counter climate change movement and conducts an important preliminary step by analyzing a cross-sectional count of the number of counter climate change organizations globally. We expand on prior data, move to a longitudinal analysis of diffusion, and elaborate on arguments for why the movement spreads globally. Drawing on expanded cross-national data on the founding dates of counter climate change organizations, we explore the role of international forces and reactionary cultural dynamics in driving the growth of these organizations around the world [10–12]. In a process akin to the “double movement” described by Polanyi [13], we suggest that the strength of a country’s commitments to protecting the natural environment generates reactionary and oppositional forms of mobilization. In other words, the rapid expansion of climate change discourse over the past several years has done more than just facilitate pro-environmental activities and outcomes; it has also reinforced stronger forms of group identification and activism among those opposed to the climate change movement [11, 14, 15]. As Zald and Useem [12: pp. 247–8] note, “by advocating change, by attacking the established interests, by mobilizing symbols and raising costs to others, movements create grievances and provide opportunities for organizational entrepreneurs to define countermovement goals and issues” (see also Mottl [16]).

We test this proposition through a series of logistic regression models for panel data on 162–164 countries from 1990 to 2018. Our results indicate that countries with stronger economic or political interests for attacking climate change discourse (e.g., those countries with higher greenhouse gas emissions per capita, higher oil rents, or higher levels of industrial activity) are not more likely to see counter climate change organizations emerge. Instead, counter climate change organizations are most likely to develop in countries with more extensive policies and structures oriented toward protecting the environment. These findings have broad implications for understanding ongoing resistance to climate change discourse and policies [17], and they speak to debates about movements that attack the legitimacy of the international liberal institutions [5, 11, 18, 19].

### Environmental institutions and the spread of counter climate change organizations around the world

Over the past several decades, the world has seen substantial institutional and discursive shifts that increasingly emphasize the importance of protecting the natural environment. For example, by the year 2000 most countries around the world had adopted national ministries of the environment, environmental impact assessment laws, or had seen the growth of a vibrant array of environmental non-governmental organizations [20, 21]. At the global level, a thick institutional infrastructure made up of international treaties (e.g., Kyoto Protocol, or the Paris Agreement of 2016), inter-governmental organizations (e.g., the Intergovernmental Panel on Climate Change [IPCC] or the United Nations Environment Programme), and international conferences (e.g., Rio Conference of 1992, or the United Nations Conference of the Parties) has emerged to construct what some have called a global “environmental regime” [22, 23]. Over time, these institutions have increasingly come to focus on climate change as the most important global social problem of the contemporary period: A long line of research has emphasized how these institutions diffuse through cultural processes that are rooted in globalizing norms, pressures, and institutional models that give these institutions their legitimacy [24].

At the same time, a substantial line of social science research has documented the rise of the “counter climate change movement” [1, 25–27]. Much of this literature has focused on the role of conservative think tanks, conservative philanthropists, and the fossil fuel industry in fueling skepticism about climate change to protect their economic or political interests. In a country like the United States, for example, high polluting actors have much to lose from large-scale policy shifts oriented toward combating climate change; these actors might therefore support efforts to fuel doubt about climate change science, produce policy studies that deny the severity of climate change, or lobby politicians to create policies that support their interests [25, 28].

Our argument emphasizes the role of culture and identity in connecting (a) the institutionalization of climate change discourse in national and international institutions, and (b) the spread of counter climate organizations around the world. As prior research has shown, the infusion of global environmental discourse into state institutions has gradually re-organized societies around norms of sustainability: for example, it has created more external pressures for countries, organizations, and individuals to act in pro-environment ways, and it has empowered new actors to advocate for pro-environmental causes [22, 24]. However, as climate change discourse becomes increasingly salient in the public sphere, oppositional groups have also formed grievances and developed adversarial goals and identities in reaction to the same set of issues, which is a key precursor to mobilization [10, 29]. Our approach is thus cultural in that it conceptualizes collective frames, narratives, and cognitive schema as the basis for the formation of identities and ideologies [30–33].

Our argument about the counter climate change movement emphasizes that the institutionalization of environmental sustainability norms around the world can also generate oppositional identities and discourse that emerge in reaction to it. For example, the presence of a perceived external threat to a group’s identity should lead to stronger perceived similarities and coordination between organizations that are otherwise dissimilar from each other [14, 34, 35]. Many of the organizations that constitute the counter-climate change movement are from a variety of sectors and have a wide range of goals, but they coalesce into a network or movement around a shared goal and identity to combat climate change policies and activity. Some organizations that make up the movement are libertarian foundations or think tanks that espouse the importance of Hayekian free markets and individual liberties for shaping what they see as free and prosperous societies (e.g., the Adam Smith Institute in the United Kingdom, or the Friedrich Naumann Foundation for Freedom in Germany). Some organizations that are part of the movement are oriented toward advancing the role of “real” or “sound” science in society, including how climate change science should be interpreted and used to make policy decisions; for example, the “Friends of Science” is an organization in Canada founded in 2002 in order to “to challenge the questionable science and destructive economic impacts inherent in the politically inspired Kyoto Protocol” [36]. In the United States, agricultural organizations are also well-represented (e.g., the American Sheep Industry Association, or the American Feed Industry Association), and many other organizations are from the industrial sector intended to protect the interests of the oil, gas, and coal industries. What unites this eclectic group of organizations is a shared identity that develops *in reaction to* the presence of a perceived common threat (climate change policies and activism) that they collectively mobilize against. In an analysis of shared hyperlinks among counter climate change organizations, McKie [9] found cohesive subgroups and substantial use of blogs for sharing information. Related, many groups attend the Heartland Institute’s annual “International Conference on Climate Change”, in its 15^th^ year in 2023. The conference brings together hundreds of high-level climate skeptics and those resistant to climate policy from across academia, government, business, and civil society.

Climate change discourse comes in many forms (e.g., media coverage, educational programs), but state action is a particularly important actor for triggering oppositional reactions [21]. As a start, states that enact climate change policies likely generate awareness of climate change issues among oppositional actors more broadly than a scattered set of pro-environmental activists or civil society organizations. Moreover, the state’s ability to create and enforce climate change laws and policies also has the potential to give the issue more “teeth”: top-down regulations could increase costs for some interest groups or disrupt longstanding practices or ways of life for others, activating grievances and opposition among those affected. Finally, state actions provide a focused target for oppositional movements to attack. Our argument suggests that counter climate change organizations should thus emerge *in reaction to* these kinds of state environmental policies that lead to the formation of oppositional identities, goals, and collective mobilization.

Overall, our argument thus suggests a theoretical process that identifies: (a) Why we expect the counter climate change movement to emerge in reaction to the global sweep of climate change discourse and institutional structures, (b) How this reaction is shaped by an underlying cultural process (e.g., organizations that emerge in reaction to perceived threats to their own identities and lead to the development of a shared identity in opposition to climate change policies and activity), and (c) How these reactionary and cultural processes shape the link between the spread of these institutional structures in countries around the world and the emergence of counter climate change organizations. In contemporary discourse, the global framing of climate change as a social problem creates a political opportunity structure that catalyzes the counter climate movement and allows the movement to gather legitimacy around the world. Over the past several decades, the formal success of the environmental movement has generated multiple institutional structures around the world that are oriented toward protecting the natural environment [20, 22, 23]. The mandates of these institutions aimed at climate change mitigation and adaptation take a broad and sweeping character that is meant to mobilize the entire world. And because climate issues are constituted as a global problem, rather than a product of the idiosyncratic concerns of countries that are economically dependent on fossil fuels, the counter climate change movement is similarly able to move from one society to another [37–39].

### Propositions and empirical implications

The foregoing discussion suggests the following proposition: *the strength of a country’s domestic commitments to protecting the natural environment is associated with the emergence of counter climate organizations in countries around the world*. To operationalize this proposition, we draw on our discussion in the previous section to identify several kinds of institutional structures we expect to shape counter climate change activity. We measure the strength of a country’s commitments to protecting the natural environment in several ways: (a) The number of international environmental agreements in force in a particular country [40], (b) The age of a country’s environmental ministry, if one exists [41], (c) The number of domestic environmental organizations in a country at a given point in time [42], and (d) The number of climate change mitigation laws or policies that are enacted in a given year [40]. We measure the separate effects of these country-level variables on the existence of counter climate change organizations; in a final set of analyses, we also combined these variables into an index that captures the overall strength of a country’s commitments to protecting the natural environment (α = 0.78).

The existence of counter climate change organizations in a given country should also be positively associated with the overall number of countries in the world that have ever had a counter climate change organization. As more of these types of organizations emerge in different countries around the world over time, we expect the counter climate change movement to gain legitimacy and cause the process of diffusion to accelerate [43]. To capture the global dimension of the counter climate movement over time, we also include a variable that measures the cumulative number of countries in the world that have ever had a counter climate change organization in our models. This variable is highly correlated with time (r = 0.99).

### Alternative arguments

In our analyses, we control for several additional factors that may be associated with our independent and dependent variables.

First, political economy arguments would expect countries that have stronger domestic economic or political interests in maintaining a carbon-based energy regime to be more likely to have counter climate change organizations [27, 44]. For example, countries more dependent on oil revenues, carbon-based forms of energy production, or industrial activity as a source of economic development may be more likely to resist broad and sweeping narratives about climate change at the global level, given that these narratives present a direct threat to their economic prosperity. In our analyses, we measure a country’s domestic economic or political interests in several ways: as a country’s oil rents as a proportion of its total GDP, total greenhouse gas emissions per capita, and a country’s levels of industrial activity (as % of GDP). All of these variables are drawn from the World Bank’s World Development Indicators [45].

Second, prior research on civil society emphasizes that a country’s level of economic development or level of democratization could be positively associated with its capacity to develop civic associations, including counter-climate change organizations [21, 46, 47]. For example, higher levels of economic development could provide individuals with greater resources, skills, or capacities to found a wide array of civic organizations, while democratic institutions create political conditions that enable and support free associations to form. We measure economic development using a standard variable for GDP per capita (logged to reduce skewness) [45], and we measure a country’s level of democratization using an index of electoral democracy from the Varieties of Democracy dataset (where a 0 indicates low levels of electoral democracy, and a 1 indicates high levels) [48].

Finally, both the growing number of counter climate organizations in countries around the world and the strength a country’s domestic commitments to protecting the natural environment may be correlated with institutional factors that encourage societal rationalization more generally. For example, a country’s levels of domestic associational life (in general) and pro-environmental organization (in particular) are both strongly correlated with its linkages to international non-governmental organizations, which provide agendas, resources, and organizational models that allow domestic organizations to thrive [21, 49]. Countries with states that have expanded social responsibilities may also be more likely to develop more domestic organizations committed to a wide array of perceived social problems in general, including those that are part of the counter climate change movement. In our analyses below, we created an index comprised of three variables that identify: (a) A country’s linkages to international non-governmental organizations (log) [50], (b) The number of domestic non-environmental organizations in a country in a given year (log) [42], and (c) The number of social ministries a country has established by a given year related to education, welfare, labor, and health (ranging from a minimum of 0 to a maximum of 4) [41, 51]. We took the z-scores for each variable and added them together to create an index (α = 0.79). This variable is highly correlated with all of the independent variables that measure the strength of a country’s environmental movement (r = 0.31–0.76, see A2 Appendix in S1 File).

Table 1 summarizes our key set of arguments and measures in our analyses. A2 Appendix in S1 File provides a correlation matrix of the independent variables used in our analyses, and A3 Appendix in S1 File provides descriptive statistics.

**Table 1:** Summary of argument and key variables.

Argument	Measure	Data sources
**Argument #1**: The strength of the domestic environmental movement is associated with the rise of counter climate change organizations.	Num. international environmental agreements in force in country i by time t Age of a country’s environmental ministry by time t Num. domestic environmental organizations (log) in country i by time t Num. climate change mitigation laws and policies enacted in country i by time t	Quality of Government Environmental Indicators (Povitkina et al 2021) Statesman’s Yearbook (various years) Gale Handbook of Associations (Gale 2021)
**Argument #2**: Counter climate organizations diffuse more rapidly as the movement spreads to other countries and gains legitimacy.	Num. countries in the world that have a counter climate organization by year t	Counter climate change organizations dataset
**Alternative argument #1**: The rise of counter climate change organizations is shaped by economic or political interests.	Oil rents (% GDP) Greenhouse gas emissions per capita Industrial activity (as % of GDP)	World Development Indicators (World Bank 2024)
**Alternative argument #2**: Counter climate change organizations are more likely to emerge in more economically or politically developed countries.	GDP per capita (log) Democracy score	World Development Indicators (World Bank 2024) Varieties of Democracy dataset (Coppedge et al 2024)
**Alternative argument #3**: The rise of counter climate orgs occurs in institutional contexts that encourage rationalization in general.	Num. linkages to international non-governmental organizations linkages (log) Num. domestic non-environmental organizations in country i, time t (log) Num. social ministries related to education, welfare, labor, and health established in country i by year t	UIA Yearbook of International Organizations (UIA, various years) Gale Handbook of Associations (Gale 2021) Statesman’s Yearbook (various years)
**Argument #1**: The strength of the domestic environmental movement is associated with the rise of counter climate change organizations.	Num. international environmental agreements in force in country i by time t Age of a country’s environmental ministry by time t Num. domestic environmental organizations (log) in country i by time t Num. climate change mitigation laws and policies enacted in country i by time t	Quality of Government Environmental Indicators (Povitkina et al 2021) Statesman’s Yearbook (various years) Gale Handbook of Associations (Gale 2021)
**Argument #2**: Counter climate organizations diffuse more rapidly as the movement spreads to other countries and gains legitimacy.	Num. countries in the world that have a counter climate organization by year t	Counter climate change organizations dataset
**Alternative argument #1**: The rise of counter climate change organizations is shaped by economic or political interests.	Oil rents (% GDP) Greenhouse gas emissions per capita Industrial activity (as % of GDP)	World Development Indicators (World Bank 2024)
**Alternative argument #2**: Counter climate change organizations are more likely to emerge in more economically or politically developed countries.	GDP per capita (log) Democracy score	World Development Indicators (World Bank 2024) Varieties of Democracy dataset (Coppedge et al 2024)
**Alternative argument #3**: The rise of counter climate orgs occurs in institutional contexts that encourage rationalization in general.	Num. linkages to international non-governmental organizations linkages (log) Num. domestic non-environmental organizations in country i, time t (log) Num. social ministries related to education, welfare, labor, and health established in country i by year t	UIA Yearbook of International Organizations (UIA, various years) Gale Handbook of Associations (Gale 2021) Statesman’s Yearbook (various years)

### Data, dependent variable, and methods

Research on the counter climate change movement now goes back more than twenty-five years [52]. Substantively, these groups cover a range of forms of counter climate beliefs, including rare instances of outright denial of climate change (more frequent in the 1980s and 1990s), but more commonly casting doubt on the degree of climate change or whether humans are the cause, casting doubt on the harms (or even suggesting many benefits), and downplaying the consequences by claiming that economic development or other priorities are far more important [8]. Studying the participants of this movement has the features of a “hidden” or “hard-to-reach” population [53]; the links between organizations and participants are opaque (and sometimes purposefully obscured) and participants consist of several subgroups. However, over several decades, a committed group of scholars and activists have built up and made available lists of climate change organizations, which we draw on for our work [1, 4, 8]. Drawing on previous research, we compiled a cross-national and historical dataset on counter-climate change organizations from the following sources: the Heartland Institute’s International Conference on Climate Change (2008–2022), the Climate Disinformation Database, and organizations identified by previous research [7, 8, 54]. We also consulted lists compiled by the Corporate Europe Observatory, the Cooler Heads Foundation, Mother Jones, the 2018 Porto Climate Action Conference, and the Union of Concerned Scientists.

In our data, counter climate change organizations are identified by our sources as having actively participated in counter climate change activities; for example, they attended or sponsored past editions of the Heartland Institute’s International Conference’s on Climate Change, or they were identified by key experts in this area. Our list of organizations focuses specifically on civil society and non-profit organizations (including think tanks, research institutes, advocacy groups, trade associations, foundations, professional associations, and university-affiliated institutes). We exclude for-profit firms and government agencies from our list. We also excluded organizations that do not directly engage in counter climate change activities as a key goal, though they might indirectly contribute to supporting the ecosystem of counter climate change organizations (for instance, consulting firms that consult for established counter climate change organizations). For each organization identified through the sources above, we collected the following information: (a) Official name, (b) Country where the organization was based or registered, (c) Official website (either active or archived), (d) Mission statement, and (e) Founding year. In total, our dataset includes 548 organizations across 51 countries.

In a best-case scenario, we would be able to identify the exact year each organization in each country started to engage in counter-climate change activity, and then conduct an event history analysis (cf. Bromley [55] on legal restrictions on foreign funding to NGOs). However, a number of organizations in the data are conservative think tanks or associations with generalist missions founded early in the 20^th^ century before the emergence of the counter climate change movement. For example, the American Petroleum Institute, the Koch Family Foundations, and the Heritage Foundation are each identified as part of the counter climate change movement, but they have founding dates of 1919, 1932, and 1973, respectively; long before “climate change” was part of social discourse. Our efforts to contact organizations quickly revealed it is implausible to obtain an exact date for their entry into counter climate action, making it necessary to use their year of founding as a proxy for engagement in counter climate change activities.

To address the issue of organizations being founded prior to the emergence of the counter climate change movement, we restrict our analyses to the years 1990 to 2018. The counter climate change movement is largely seen as gaining traction after the Global Climate Coalition was initiated in 1989, which was the first and largest domestic coalition in the United States to oppose climate change after the creation of the IPCC in 1988 [1, 8, 56, 57]. This coalition was particularly successful in giving the counter climate change movement legitimacy because of its size, the actors involved, and its ability to pour resources into fueling organized opposition [57]. Prior to this, it is unlikely that organizations in our data are actively involved in counter climate change activities, given that there was little to mobilize against. We therefore assume that organizations founded after 1990 in our data are part of the counter climate change movement; we also assume that organizations founded before this date are not actively involved in counter-climate change activities until after 1989 (see A1 Appendix in S1 File for a list that identifies the first year a counter climate change organization is founded in a given country).

In our analyses, the dependent variable is measured as a dichotomous variable that identifies whether a country has ever seen a “counter climate change” organization founded by year *t*. We treat the founding year of an organization as the year it becomes “counter climate change” during this time period; any organization that is founded before 1990 is assigned 1990 as its founding date. When we use this approach, 25 countries have a counter-climate change organization by 1990, out of the 51 countries that have at least one organization by 2022. Given a number of countries that have an organization by the starting point of our analyses, we are unable to measure our dependent variable as a duration outcome through an event history analysis: these countries would be considered left-censored in a survival analysis, and would bias our coefficients given that they would not be included in our analyses.

We instead use logistic regression models for panel data with random effects to estimate the effects of our independent variables on whether a country has ever had a counter climate change organization. Logistic regression models are appropriate for this set of analyses, given that our dependent variable follows a binomial distribution [58]; our coefficients are reported as log odds, where a country’s probability of ever having a counter climate change organization is transformed through a logit link function. We use random effects panel regression models, which exploit both within- and between-country variation in our dependent and independent variables; fixed effects models for panel data rely only on within-country variation over time, but are not appropriate for this set of analyses because many of the countries in our data do not vary in the dependent variable over time (e.g., many countries never have a counter climate change organization) [59, 60]. All independent and control variables are lagged by one year; our results are similar when alternative lag times are used.

## Results

**Fig 1:**
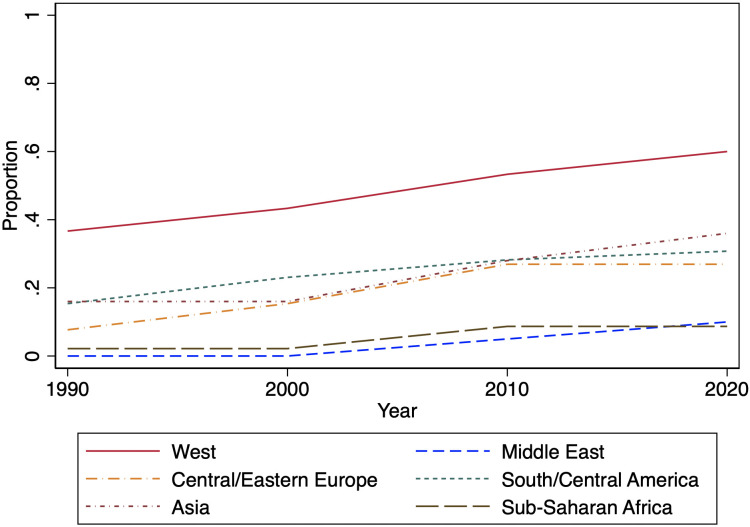
Proportion of countries around the world with at least one counter-climate organization.

Fig 1 plots the proportion of countries around the world where a counter climate organization has ever been founded from 1990 to 2022. By 2022, 26 percent of countries in the world have at least one such organization. As our data show, most of the counter climate organizations that have emerged over this period are based in the industrialized West; however, a non-trivial proportion of countries in South/Central America, Asia, and Central/Eastern Europe have also had at least one of these organizations during this period. In our data, the U.S. is the home to by far the largest number of these organizations in the world: 350 organizations, out of 548, are from the United States (over 60 percent). Other countries in the Anglosphere also have relatively high counts of counter climate change organizations, compared to other countries: among the total count of organizations in our data, 34 are in the United Kingdom, 19 are in Canada, and 16 are in Australia.

Table 2 presents the main results of our logistic regression models from 1990 to 2018. Our regression analyses end in the year 2018, rather than 2022, because of limited data availability on some of our independent variables. Across each of our models, we find consistent evidence for our argument about the role of reactionary dynamics in shaping the diffusion of counter climate change organizations around the world. In particular, the number of international environmental agreements in force (Model 2), the age of a country’s ministry of environment (Model 3), the number of domestic environmental organizations (Model 4), and the cumulative number of climate change mitigation laws and policies adopted in a given year (Model 5) are all positively associated with whether a country has ever had a counter climate organization (p < 0.05 in all models). In Model 6, an index that captures the strength of a country’s environmental movement is also positively and significantly associated with having a counter climate organization; this result is also robust when our measure of climate change mitigation laws and policies is excluded from the index (given its smaller sample size than our other measures). Across each model, the coefficient for our variable that identifies the number of countries in the world that have a counter climate organization is also positively and significantly associated with the diffusion of these organizations to other countries. In supplementary analyses, we also found a positive and significant association between the proportion of countries in a given region (rather than the world) that have ever had a counter climate organization and the emergence of a counter climate organization in a country.

**Table 2:** Random effects panel logistic regression models: Whether a country has ever had a counter climate change organization, 1990–2018.

	(1)	(2)	(3)	(4)	(5)	(6)
GDP per 10,000 capita (logged)	0.41	-1.10	-0.93*	1.99+	-1.36*	-2.83+
	(0.52)	(0.69)	(0.41)	(1.03)	(0.63)	(1.60)
Industry (% GDP)	-0.05	-0.04	0.05	-0.03	0.12+	0.07
	(0.05)	(0.06)	(0.05)	(0.08)	(0.06)	(0.13)
Democracy score	8.34***	5.77*	6.26***	6.35+	5.02**	3.60
	(1.94)	(2.34)	(1.70)	(3.27)	(1.87)	(6.34)
Oil rents (% GDP)	-0.45***	-0.41***	-0.39***	-0.23	-0.56***	-0.90**
	(0.13)	(0.12)	(0.09)	(0.17)	(0.16)	(0.29)
Greenhouse gas emissions per capita	-2.38+	-0.49	-1.93+	-2.12	-0.56	0.04
	(1.26)	(1.35)	(0.99)	(2.49)	(1.41)	(2.28)
# of countries w/ counter-climate org	0.47***	0.36***	0.37***	0.42***	0.34***	0.34*
	(0.04)	(0.07)	(0.03)	(0.11)	(0.05)	(0.14)
Rationalization index^1^	3.07***	4.10***	3.29***	4.40***	4.50***	8.43***
	(0.33)	(0.44)	(0.38)	(0.54)	(0.50)	(1.26)
Int’l env. agreements in force		0.04***				
		(0.01)				
# of domestic environmental orgs (log)			1.62**			
			(0.53)			
Age of environment ministry				0.28***		
				(0.08)		
# of climate change mitigation laws/policies					0.45***	
					(0.10)	
Commitments to environmental protection index^2^						2.50***
						(0.62)
Constant	-39.91***	-24.18***	-25.29***	-66.26***	-23.72***	-36.50**
	(4.05)	(6.04)	(3.02)	(6.64)	(4.51)	(12.26)
lnsig2u	5.75***	6.06***	5.01***	6.48***	5.19***	7.19***
	(0.25)	(0.31)	(0.25)	(0.21)	(0.25)	(0.14)
N	4,313	4,307	4,313	4,313	2,857	2,857
Countries	164	164	164	164	162	162
Df	7	8	8	8	8	8
λ^2^	713.01	450.16	430.31	689.79	362.92	376.60

**Notes**: Standard errors in parentheses.

^+^ p<0.10

* p<0.05

** p<0.01

*** p<0.001.

All independent/control variables lagged 1 year.

^1^ Rationalization index is comprised of three variables that identify: A country’s linkages to international non-governmental organizations (log), The number of domestic non-environmental organizations in a country in a given year (log), and the number of social ministries a country has established by a given year related to education, welfare, labor, and health (minimum of 0, maximum of 4). We took the z-scores for each variable and added them into an index (α = 0.79).

^2^ The commitments to environmental protection index is made up of four variables: Number of international environmental agreements in force (QOQ) in a given year, age of a country’s ministry of environment (Statesmen’s Yearbook), number of domestic environmental organizations (Gale), and cumulative number of climate change mitigation laws and policies in a given year. We took the z-scores for each variable and added them to create an index (α = 0.78). Given that the sample size for our data on climate mitigation laws/policies is smaller than for our other variables, we also tested this index with only the first three variables; the results are unchanged.

**Fig 2:**
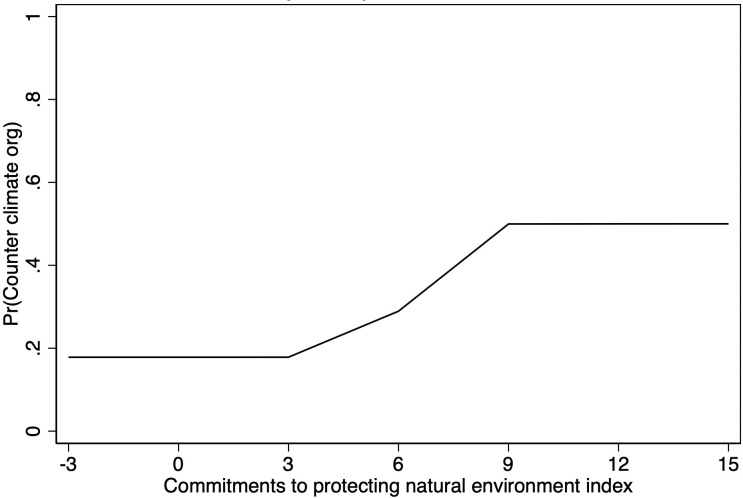
Predicted probability that a country has a counter climate change organization, by the strength of its domestic environmental movement. **Note**: Figure is based on the regression results from Table 1, Model 6.

Fig 2 plots the predicted probability that a country will have a counter climate organization by the strength of its commitments to protecting the natural environment (based on regression estimates from Model 6), when all other variables in the model are held at their means. These results draw a clear link between the strength of a country’s commitments to protecting the natural environment and the growth of counter climate organizations around the world: countries that have the strongest commitment to environmental causes (based on our index) have a 50 percent predicted probability of having a counter climate organization. By contrast, countries with the weakest commitments to environmental causes in our data have less than a 20 percent predicted probability of having a counter climate organization.

Our results do not show consistent support for arguments that emphasize the importance of economic development and political/economic interests in shaping the diffusion of the counter climate movement globally. For example, a country’s GDP per capita is inconsistently associated with the founding of counter climate change organizations. The coefficients that capture a country’s oil rents (as % of GDP) indicate a significant association with the emergence of counter climate change organizations, but the signs of the coefficients are in an unexpected direction: countries with higher oil rents are the *least* likely to have a counter climate organization. A country’s greenhouse gas emissions per capita and industrial activity are also not significantly associated with the emergence of counter climate change organizations. These findings suggest the importance of treating the counter climate change movement as shaped by more then a country’s domestic economic or political interests; instead, it is shaped by reactionary and oppositional dynamics that produce counter-movements in the presence of state policies and institutions committed to protecting the natural environment.

Finally, it is also worth noting that our overall index of rationalization is consistently positively associated with the founding of counter climate organizations in countries around the world. Many other variables that are related to a country’s integration into liberal world society (e.g., tertiary enrollment ratios, international trade treaties, economic globalization, or human rights treaties) are also positively and significantly associated with the rise of counter climate organizations; given that they are highly correlated with this index, however, including them in our models would create issues of multicollinearity. This result implies that the same kinds of organizational strategies or cultural scripts that are propagated by international non-governmental organizations to support liberal causes can also be appropriated by oppositional groups to undermine these causes [5, 61]. For example, climate change denialists can claim to oppose climate change discourse on “scientific” grounds, or as impingements on individual rights [28, 52]. They can also appeal to their own educated “experts” to legitimate their contrarian claims, or they can organize conferences to spread information about the climate denialist movement. For example, the Heartland Institute’s conference to undermine climate support includes “science” and “policy” tracks, just as a conference working towards adaptation and mitigation might. While many cross-national studies on the expansion of domestic associational life have emphasized the rise of civic organizations that fit the ideals of liberal world society over the postwar period (e.g., development, education, environment, gender equality, science, among others), alternative ideologies may become more prominent over time as the legitimacy of the liberal international order continues to be attacked [6, 18, 19, 55]. We discuss this issue in more depth in our concluding reflections.

### Additional analyses and robustness checks

In supplementary analyses, we explored several additional possible arguments.

First, given that most of these organizations are hosted in the United States, we tested whether countries that are more strongly tied to the US, either through USAID support or through trade dependence with the US, were more likely to have counter climate change organizations. Neither of these variables were significantly associated with our dependent variable. We also checked the robustness of our results when the US is excluded from the analyses, given that it is possible that the country’s outlier status may skew our results; our estimates remain unchanged.

Second, some have argued that rapid globalization in the 1990s generated rising economic inequalities in the developed world, leading to an erosion of trust in established global elites and institutions [62; see 63, 64 for reviews]; given this, it is possible that counter climate organizations are just one dimension of a more general form of backlash against globalization and increasing income inequality. Drawing on data from the Standardized World Income Inequality Database [65], we tested whether countries that have higher levels of income inequality are more likely to adopt counter climate organizations. We did not find support for this hypothesis; given that this variable reduced our sample size by a sizeable amount in our regressions, we chose not include this variable in our final set of analyses.

Third, theories of social movements emphasize the importance of political allies and political opportunities in shaping social movement activity. Much of the opposition to climate change action has come from political right [26, 52, 66], particularly given that the scale of climate change as a global social problem entails substantial societal shifts away from the status quo. We explored whether the ideology of a country’s political leader shapes the emergence of counter climate change organizations in two ways. First, we tested whether countries with political leaders from the right are more likely to see counter climate change organizations emerge, using data from the Inter-American Development Bank’s Database of Political Institutions [67]. Second, we examined whether countries with populist leaders from the right or left were more likely to have counter climate change organizations, using data on populist leaders around the world collected by Cole and Schofer [68]. Given that the counter climate change movement is in large part a form of resistance to broad and sweeping narratives about climate propagated by global elites, it is possible that counter climate change organizations share links with the resurgence of populist movements around the world [63, 64]. Neither of these measures, however, yielded any significant results. As these variables in our analyses reduced our sample size and did not improve model fit, we opted not to include them in our final set of models.

Finally, we also checked how our results changed when we measured the dependent variable as a count of the number of counter climate change organizations a country has at a given point in time, using Poisson regression models for panel data [58]. In these analyses, we top-code the number of organizations in the US as 34 (the second highest number of organizations in a country in our data) because it is a significant outlier and could skew our results. The main concern for our paper is to understand when countries see counter climate organizations emerge, rather than to understand the degree of organizational activity around counter climate change issues; nonetheless, our argument should be relevant to both questions. Our findings are, by and large, similar to those reported above: we see consistent positive coefficients for our variables that capture the strength of a country’s commitments to protecting the natural environment on the number of counter climate change organizations. The coefficient for our variable that captures the global diffusion of these organizations is only inconsistently significant, however. The results for our control variables are also mostly unchanged, except that in some cases countries with more greenhouse gas emissions per capita are significantly more likely to have higher counts of counter climate change organizations. The findings of our cross-sectional count analysis are also generally similar to McKie [7], who reports that pro-environment indicators (in her case measured by the number of university climate centers, environmental NGOs, and protected land) all increase the number of predicted counter climate change organizations; while resource-based arguments such as oil rents, GDP per capita, ecological footprint, and greenhouse gas emissions have inconsistent results (some are negative, some are positive, others are not significant).

## Conclusion

Broadly, the increasing availability of cross-national and longitudinal data on counter climate organizations creates many new avenues for research that could provide insight into anti-climate action. For example, one could examine whether counter climate change organizations affect environmental outcomes at the country level: for example, do countries with more of these organizations generate more environmental degradation and fewer climate change policies? These effects could also change over time, as the counter climate change movement’s tactics shift from denying that climate change exists toward claiming that climate change solutions won’t work [69]. In addition, anecdotal accounts in a position paper produced by the think tank Climate Social Science Network suggests there may be systematic differences between counter climate change organizations in developed and less developed countries [70]. Further, counter-climate change activism may unfold in waves within countries, perhaps initially driven by industry interests and subsequently driven by more diffuse anti-liberal or anti-progressive cultural movements; these two forms may also blend in forms that could be uncovered through qualitative case work. These potential geographic and temporal variations are of great importance for understanding the evolution and influence of the counter climate change movement.

This paper emphasizes the importance of reactionary dynamics in shaping the emergence of oppositional groups around the world, net of resource dependencies. As our analyses show, the strongest predictors of the emergence of counter climate change organizations are the strength of a country’s commitments to protecting the natural environment (particularly through the state’s adoption of institutional structures and policies). Strong state structures and policies to protect the environment makes climate change issues more salient in the public sphere, activates adversarial grievances among oppositional groups, and reinforces group identification among those opposed to the climate change movement. The cultural and reactionary processes we outline in the paper are thus mechanisms that explain the robust link between the existence of these pro-environmental domestic institutions and the emergence of its opposite–counter climate change organizations. In our analyses, these cultural factors are much more prominent than the factors that identify economic or political interests at the country level, which show inconsistent or sometimes even negative effects on the emergence of counter climate organizations.

Attending to the cultural and reactionary dynamics of the counter climate change movement is important for understanding the role of environmental institutions and policies in shaping social change. Rather than seeing pro-environmental structures and policies as entirely separate from the counter climate change movement, our emphasis on culture and identity suggests that both movements are intertwined and evolve in tandem with each other as part of a dynamic process. Our findings suggest that the success of the pro-environmental movement can create conditions that enable the counter climate change movement to grow. To understand environmental change, it is important to unpack the heterogeneous outcomes of strong pro-environmental structures and policies, and the conditions that create this heterogeneity. For example, environmental institutions can lead to clear pro-environmental change under some conditions [22, 24, 71], while under other conditions pro-environmental policies can lead to greenwashing [72–75] or even mobilize anti-environmental movements. Additional research seeking to understand the conditions under which reactionary dynamics arise would be valuable, particularly in the interplay between economic interests and the development of state policy.

More practically, our study suggests that as a routine part of the policy process it ought to be important for states to investigate potential reactionary movements that might be triggered by environmental policy. Similarly, environmental organizations may want to consider how their actions can both achieve goals and mitigate counter reactions; for example, Dolsak and Prakash [76] suggest that destructive protests (e.g., defacing art) undercut popular support for the environmental movement. Perhaps solutions that do not strengthen the cultural movement against climate action are available.

Looking more broadly, our data indicate that climate change is one arena where oppositional movements have gained traction over the past few years (for other arenas see, e.g., on LGBT+ rights, [11, 15]; on higher education, [19]; on human rights, [77]). Much of the sociological research on post-liberal oppositional movements has emphasized the role of illiberal international organizations (e.g., the Commonwealth of Independent States, League of Arab States, or Shanghai Cooperation Organization) in spreading countervailing illiberal norms and practices to member countries [55, 77, 78]. In supplementary analyses (not shown), however, we did not find that countries with more ties to illiberal international organizations were more likely to be associated with the emergence of counter climate change organizations. It is possible that the counter climate change movement does not fit neatly into a dichotomy of liberal versus illiberal movements. On one hand, our analyses suggest that counter climate change organizations are a clear oppositional reaction to the environmental movement (a core component of liberal world society). On the other hand, however, many of these organizations extol the ideals of classical liberalism: for example, free markets, individual liberties, and their role in shaping free and prosperous societies. In future work, it could also be important to distinguish between post-liberal backlash that emerges from pressures from external, global institutional models (i.e. neocolonialism), and backlash generated by institutional contradictions that create internal conflicts within liberal societies (cf. [5] on the anti-vaccination movement and how individualism generates both scientific advancement and anti-science reactions). Overall, the dynamics of reactionary mobilization and oppositional movements are important to consider for how they might shed light on effective climate action, and as part of a wider backlash against the institutions that have shaped the liberal world order.

## Supporting information

S1 File Appendices. (DOCX)
