# Supplementary material for: Globalizing opposition to pro-environmental institutions: The growth of counter climate change organizations around the world, 1990 to 2018
Source: PLoS One. 2025 Jan 22;20(1):e0315012. doi: 10.1371/journal.pone.0315012 (PMC11753699; doi:10.1371/journal.pone.0315012)
Supplement: S1 File — (DOCX) [file pone.0315012.s001.docx]

| **Appendix A1.** List of countries and the year of first founding of a counter-climate change organization | | | |
| --- | --- | --- | --- |
| **Country** | **Founding year** | **Country** | **Founding year** |
| Australia | 1990 | Turkey | 1992 |
| Belgium | 1990 | Austria | 1993 |
| Brazil | 1990 | Bulgaria | 1993 |
| Canada | 1990 | Bahamas | 1995 |
| China | 1990 | Argentina | 1998 |
| Czech Republic | 1990 | Belarus | 1999 |
| Denmark | 1990 | Georgia | 2001 |
| France | 1990 | Nigeria | 2002 |
| Germany | 1990 | Albania | 2003 |
| Greece | 1990 | Israel | 2003 |
| Guatemala | 1990 | Italy | 2003 |
| India | 1990 | Portugal | 2003 |
| Korea, Rep | 1990 | Ghana | 2004 |
| New Zealand | 1990 | Hong Kong | 2004 |
| Peru | 1990 | Mexico | 2004 |
| Philippines | 1990 | Honduras | 2006 |
| Russian Federation | 1990 | Poland | 2006 |
| South Africa | 1990 | Burkina Faso | 2007 |
| Spain | 1990 | Romania | 2009 |
| Switzerland | 1990 | Sweden | 2009 |
| Taiwan | 1990 | Indonesia | 2010 |
| United Kingdom | 1990 | Malaysia | 2010 |
| United States | 1990 | Jordan | 2012 |
| Venezuela, RB | 1990 | Singapore | 2012 |
| Chile | 1990 | Pakistan | 2013 |
| Paraguay | 1990 | Colombia | 2016 |
| Ecuador | 1991 | Ireland | 2016 |
|  |  | Netherlands | 2018 |
| **Note:** As we discuss in the paper, the counter climate change movement is largely understood to have emerged after the Global Climate Coalition was formed in 1989. In our data, we treat the founding year of an organization as the year it begins engaging in “counter climate change” activities during the time period of our analyses (i.e., 1990 to 2018); however, many organizations in our dataset were founded before 1989. To address this issue, we assign a founding date of 1990 to these organizations in our data that were founded before 1989. It is unlikely that organizations founded before 1989 were actively involved in counter-climate change activities, given that climate change was not an issue to mobilize against at the time. | | | |

| **Appendix A2**. Correlation matrix of key dependent and independent variables. | | | | | | | | | | | | |
| --- | --- | --- | --- | --- | --- | --- | --- | --- | --- | --- | --- | --- |
|  | 1 | 2 | 3 | 4 | 5 | 6 | 7 | 8 | 9 | 10 | 11 | 12 |
| (1) Counter climate org (d) | 1.00 |  |  |  |  |  |  |  |  |  |  |  |
| (2) GDP per capita (log) | 0.39 | 1.00 |  |  |  |  |  |  |  |  |  |  |
| (3) Industry (% GDP) | -0.02 | 0.13 | 1.00 |  |  |  |  |  |  |  |  |  |
| (4) Democracy score | 0.38 | 0.59 | -0.28 | 1.00 |  |  |  |  |  |  |  |  |
| (5) Oil rents (% GDP) | -0.16 | 0.04 | 0.70 | -0.35 | 1.00 |  |  |  |  |  |  |  |
| (6) Greenhouse gas emissions per capita | 0.22 | 0.70 | 0.31 | 0.24 | 0.23 | 1.00 |  |  |  |  |  |  |
| (7) Int'l env agreements | 0.42 | 0.61 | -0.12 | 0.53 | -0.09 | 0.31 | 1.00 |  |  |  |  |  |
| (8) # of env orgs (log) | 0.48 | 0.54 | -0.10 | 0.52 | -0.23 | 0.36 | 0.49 | 1.00 |  |  |  |  |
| (9) Age of env ministry | 0.16 | 0.31 | -0.16 | 0.29 | -0.12 | 0.21 | 0.52 | 0.26 | 1.00 |  |  |  |
| (10) # of climate change mitigation laws | 0.36 | 0.26 | -0.05 | 0.21 | -0.11 | 0.06 | 0.53 | 0.24 | 0.36 | 1.00 |  |  |
| (11) Commitments to environment index | 0.48 | 0.59 | -0.15 | 0.53 | -0.19 | 0.32 | 0.85 | 0.69 | 0.72 | 0.70 | 1.00 |  |
| (12) Rationalization index | 0.57 | 0.65 | -0.06 | 0.57 | -0.19 | 0.39 | 0.59 | 0.76 | 0.31 | 0.37 | 0.70 | 1.00 |
| (13) # of countries w/ a counter climate org | 0.01 | 0.04 | -0.06 | -0.04 | 0.04 | 0.00 | 0.48 | -0.04 | 0.45 | 0.51 | 0.46 | -0.05 |

| **Appendix A3.** Descriptive statistics of key variables | | | | | |
| --- | --- | --- | --- | --- | --- |
| **Variable** | **Obs** | **Mean** | **S.D.** | **Min** | **Max** |
| Counter climate org (d) | 4,313 | 0.24 | N/A | 0.00 | 1.00 |
| GDP per capita (log) | 4,313 | 8.33 | 1.44 | 5.25 | 11.63 |
| Industry (% GDP) | 4,313 | 27.76 | 11.46 | 3.24 | 86.67 |
| Democracy score | 4,313 | 0.52 | 0.27 | 0.01 | 0.92 |
| Oil rents (% GDP) | 4,313 | 3.70 | 9.31 | 0.00 | 65.16 |
| Greenhouse gas emissions per capita | 4,313 | 0.65 | 0.69 | 0.02 | 5.60 |
| Int'l env agreements | 4,307 | 178.16 | 117.03 | 0.00 | 624.00 |
| # of env orgs (log) | 4,313 | 1.20 | 1.09 | 0.00 | 5.24 |
| Age of env ministry | 4,313 | 12.64 | 11.22 | 0.00 | 47.00 |
| # of climate change mitigation laws | 2,857 | 4.03 | 4.09 | 0.00 | 29.00 |
| Commitments to environment index | 2,857 | 1.63 | 2.91 | -3.73 | 13.99 |
| Rationalization index | 4,313 | 1.09 | 1.91 | -7.42 | 5.81 |
| # of countries w/ a counter climate org | 4,313 | 39.94 | 8.94 | 25.00 | 53.00 |
